# Supplementary material for: Content-rich biological network constructed by mining PubMed abstracts
Source: BMC Bioinformatics. 2004 Oct 8;5:147. doi: 10.1186/1471-2105-5-147 (PMC528731; doi:10.1186/1471-2105-5-147)
Supplement: Additional File 2 — The original results of the above study (non-essential files are deleted to keep the file size under the limit set by BMC bioinformatics). [file 1471-2105-5-147-S2.bz2 › chilibotAdditionalFile2/dip10/573ID2164219E10940/html/CD28_CD80.html]

 


 **CD28** and **CD80** 
  
Found 551 abstracts in PubMed, retrieved 10.  
 

 What does Google say? 
 PDF only 
| .edu only 

---

**Interactive relationship** (e.g. stimulation, inhibition, etc)

**Inhibitory relationship**- Attempts at blocking the  **CD28**  B7s  **CD80**  CD86 pathway are currently underway with the receptor fusion protein, LEA29Y a second generation CTL4Aig, and humanized mAbs to CD 80  [ **CD80** ]  and CD86.  Ref: 12773967 Minerva Urol Nefrol, 2003
**Neutral relationship**- The importance of interactions of  **CD80**  CD86 and  **CD28**  in activating CD4 T cells was clear.  Ref: 12794123 J Immunol, 2003
- An effective humoral response requires bidirectional interactions between B cells and CD4 T cells, critical of which are interactions between  **CD80**  CD86 expressed on activated B cells and  **CD28**  expressed on responder CD4 T cells.  Ref: 12794123 J Immunol, 2003
- Fas ligand FasL, that has the inherent capacities to tether the T cell inhibitor FasL CD95 ligand to the surfaces of B7  **CD80**  and CD86 positive APC via CTLA 4 B7 interaction, and in so doing, to simultaneously interfere with B7 to  **CD28**  T cell activation signals.  Ref: 12794109 J Immunol, 2003

**Non-interactive relationship** (e.g. studied together, co-existance, homology, etc.)

- These results demonstrate, for the first time, a critical contribution of the  **CD28**   **CD80**  CD86 costimulatory pathway not only to natural protection against primary T. cruzi infection but also to DNA vaccine induced protective immunity to Chagas disease.  Ref: 12761091 Infect Immun< MedlineTA>Infect Immun, 2003
- In this study, we investigated the role of this costimulatory pathway in resistance to Trypanosoma cruzi infection by using  **CD28**  deficient mice and blocking antibodies against  **CD80**  and CD86.  Ref: 12761091 Infect Immun< MedlineTA>Infect Immun, 2003
- the ablation of a normal response following the addition of neutralizing anti CD86  **CD80**  Abs mirrored the response of CD4 T cells to B cells of HIV viremic patients, while the addition of exogenous  **CD28**  ligands partially restored the poor CD4 T cell response to the B cells of HIV viremic patients.  Ref: 12794123 J Immunol, 2003
- Critical Contribution of  **CD28**   **CD80**  CD86 Costimulatory Pathway to Protection from Trypanosoma cruzi Infection.  Ref: 12761091 Infect Immun< MedlineTA>Infect Immun, 2003
- The  **CD28**   **CD80**  CD86 mediated T cell costimulatory pathway has been variably implicated in infectious immunity.  Ref: 12761091 Infect Immun< MedlineTA>Infect Immun, 2003
- Melanoma patients with thinner tumors and those surviving revealed an increase of CD19  **CD80**  and CD19  **CD80**  CD86 cells, as well as a higher concentration of CD3 CD4  **CD28**  cells.  Ref: 12878358 Hum ImmunolHum Immunol, 2003
- Expression of costimulatory molecules, CD49D and  **CD28**  on T cells and  **CD80**  and CD86 on monocytes, was unchanged.  Ref: 12753529 Respirology< MedlineTA>Respirology, 2003
- Changes in the number of  **CD80** , CD86, and  **CD28**  peripheral blood lymphocytes have prognostic value in melanoma patients.  Ref: 12878358 Hum ImmunolHum Immunol, 2003
- We determined the concentration of peripheral blood lymphocytes, which expressed the  **CD80**  CD86 or  **CD28**  CTLA 4 molecules, in 38 patients with cutaneous melanoma and 27 controls.  Ref: 12878358 Hum ImmunolHum Immunol, 2003
- Expression of CD4, CD8alpha, CD40, CD86,  **CD28** , CD31 and vascular endothelial growth factor was not different between the two tumor groups but expression of CD40 ligand,  **CD80** , NK1.1 and CXCR3 was relatively lower in A11 Mig tumors.  Ref: 12792744 Oncol Rep< MedlineTA>Oncol Rep, 2003
